# Microarray Analysis of Transcriptional Responses to Abscisic Acid and Salt Stress in *Arabidopsis thaliana*

**DOI:** 10.3390/ijms14059979

**Published:** 2013-05-10

**Authors:** Yujia Liu, Xiaoyu Ji, Lei Zheng, Xianguang Nie, Yucheng Wang

**Affiliations:** State Key Laboratory of Forest Genetics and Tree Breeding, Northeast Forestry University, 26 Hexing Road, Harbin 150040, China; E-Mails: liuyujia0703@yahoo.cn (Y.L.); jixy0219@yahoo.com.cn (X.J.); Lei.zheng123@yahoo.com.cn (L.Z.); nxg_OK@126.com (X.N.)

**Keywords:** microarray analysis, *Arabidopsis thaliana*, gene expression, ABA, salt stress

## Abstract

Abscisic acid (ABA) plays a crucial role in plant responses to abiotic stress. To investigate differences in plant responses to salt and ABA stimulus, differences in gene expression in *Arabidopsis* in response to salt and ABA were compared using an Agilent oligo microarray. A total of 144 and 139 genes were significantly up- and downregulated, respectively, under NaCl stress, while 406 and 381 genes were significantly up- and downregulated, respectively, under ABA stress conditions. In addition, 31 genes were upregulated by both NaCl and ABA stresses, and 23 genes were downregulated by these stressors, suggesting that these genes may play similar roles in plant responses to salt and ABA stress. Gene ontology (GO) analysis revealed four subgroups of genes, including genes in the GO categories “Molecular transducer activity”, “Growth”, “Biological adhesion” and “Pigmentation”, which were expressed in response to ABA stress but not NaCl stress. In addition, genes that play specific roles during salt or ABA stress were identified. Our results may help elucidate differences in the response of plants to salt and ABA stress.

## 1. Introduction

Microarray technologies have become a routine tool for the analysis of transcript abundance in the life sciences [1]. For the analysis of defined stimuli such as hormone or abiotic stress treatments, transcriptional profiling has been particularly informative [2]. The microarray analysis of *Arabidopsis thaliana* genome has provided a powerful and widely used method to research the effects of various gene expressions [3,4].

Salinity is the major environmental factor that limits plant growth, which affects the quantity and quality of crops produced [5,6]. According to some studies, nearly 40% of irrigated land worldwide is under quite serious threats of salinization, and the problem of soil salinity is increasing over time [7]. A few plants have developed special organs to improve salt tolerance through active secretion of salt and passive rejection of salt to avoid salt stress [8]. High salinity causes both ionic and osmotic stresses leading to reduced growth rates and eventually to plant death [9]. Salt stress is composed of different stresses that induce an overlapping pattern of gene expression. For example, in a survey of 8,100 *Arabidopsis* genes, around 2400 genes were observed as having a common expression in response to salt, osmotic and cold stress treatments [10]. In other wards, a common set of signal transduction components is triggered during many stress responses [11]. Most commonly, high Na^+^ and Cl^−^ cause the salt stress. NaCl was able to not only induce the osmotic stress [12], but also disturb ion homeostasis and toxicity [13]. In the past few years, microarray analyses of *Arabidopsis thaliana* and rice plants identified hundreds of stress responses under stress treatments [14–18].

ABA is defined as a stress hormone because of its rapid accumulation in response to stresses and its mediation of many stress responses that help plants adapt to environmental challenges [19–21]. Over the past few decades, numerous ABA signaling components, including ABA receptors, have been identified [22–24], and the relationship between ABA accumulation and plant tolerance to osmotic, salt, and chilling stresses has been corroborated [25,26]. Stress-responsive genes can be expressed either through ABA-dependent or ABA-independent regulatory pathways in plants [27,28]. Hence, comparing differences in gene expression is an important method for clarifying differences between ABA-dependent and ABA-independent plant responses to stress. The promoter domains in all ABA-responsive genes contain the ABA-responsive element ABRE [29,30]. Various transcription factors regulate ABA-responsive gene expression [25,31], such as DREB2A/2B, AREB1, RD22BP1 and MYC/MYB, through interactions with their corresponding cis-acting elements, *i.e.*, DRE/CRT, ABRE and MYCRS/MYBRS, respectively [32,33]. However, WRKY transcription factors were regulated by ABA treatment, and ABO3 that antagonize the SnRK2-dependent AREB activation [23].

In many ways, ABA acts as a mediator in the whole-plant response to salt stress [34]. ABA contributes to the increase of xylem water potential, as well as water uptake, in plants subjected to salt stress [35]. According to current understanding, ABA is the long-distance signal that communicates water stress from the root to the shoot [36]. Nevertheless, the point was not supported in the study of [37], indicating that the shoot response to limited soil water supply is not affected by the capacity to generate ABA in the root; however, the response does require ABA biosynthesis and signaling in the shoot. Recently, ABA was found to be a vital cellular signal that mediates the expression of a number of salt-responsive genes. For example, the salt-responsive genes AtNHXl and AtNHX2 are induced by ABA [38]. In addition, salt stress not only induces ABA-mediated gene expression, but it also promotes ABA biosynthesis by transcriptional regulating ABA-biosynthetic genes [39,40]. However, not all salt-responsive genes are affected by ABA, which indicates that the expression of salt-responsive genes can involve ABA-dependent and ABA-independent pathways [41]. The accumulation of RD29A and proline transcripts are regulated in both ABA-dependent and ABA-independent manners [42]. In the present study, we investigated differences in gene expression in *Arabidopsis* subjected to NaCl *vs.* ABA treatment using an Agilent oligo microarray. Genes that play common or specific roles in salt or ABA stress were identified. Our results may help elucidate differences in plant responses to salt and ABA stress.

## 2. Results and Discussion

### 2.1. Genes Expressed in Response to Salt Stress

To investigate changes in *Arabidopsis* gene expression in response to salt stress, gene expression in plants treated with 150 mM NaCl for 6 h before harvest, were studied alongside the control plants, using Agilent GeneChip microarray analysis. With *p*-value > 0.05 and a threshold of twofold change, a total of 144 and 139 genes that were upregulated and downregulated, respectively, in response to salt stress were identified (Supporting Information Table S1). Among the differentially expressed genes, some genes encoding proteins in the same family were enriched under salt stress conditions (Supporting Information Table S1), including genes encoding zinc finger proteins, bHLH family proteins, NAC transcription factors, LTP family proteins, TCP family transcription factors, miscellaneous RNA (misc RNA) and heat shock proteins, indicating that these genes may play important roles in mediating stress tolerance.

Of these differential expression genes, we identified two drought-responsive genes, rd22 and rd26, which were well known as stress-inducible genes [43,44]. Furthermore, there was a gene encoding an oxygen-binding protein which is involved in oxidative stress response [16]. In addition, we also obtained a Serine carboxypeptidase S10 family protein which is regarded as a salt stress-specific marker gene in *Arabidopsis thaliana* [2]. These results are consistent with our previous finding that a common set of signal transduction components are triggered in many stress responses. In our experiment, however, the number of salt-responsive genes is significantly lower than that in the studies of Kreps *et al.* [10] and Kilian *et al.* [11].

**Figure 1:**
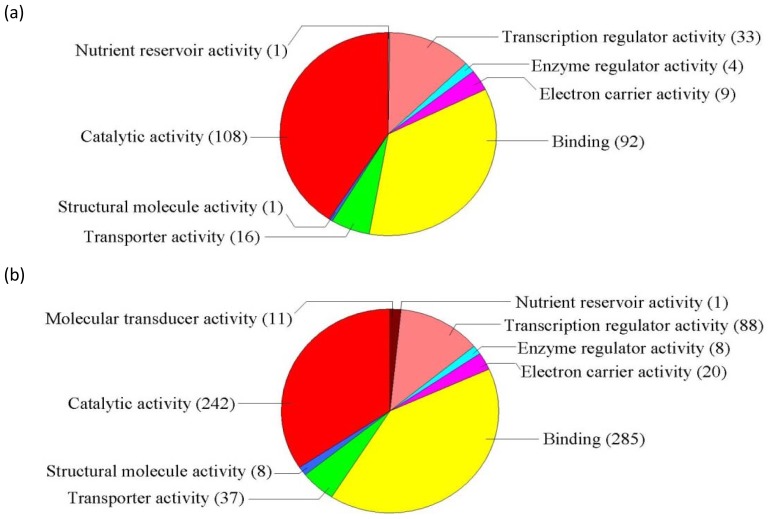
GO term pie chart of “Molecular function” for NaCl- and ABA-stressed genes. (**a**) Indicates GO term pie picture of “Molecular function” under NaCl-stressed genes; (**b**) Indicates GO term pie picture of “Molecular function” under ABA-stressed genes.

**Figure 2:**
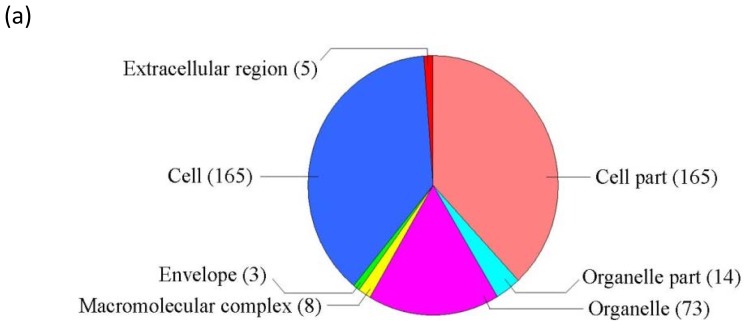

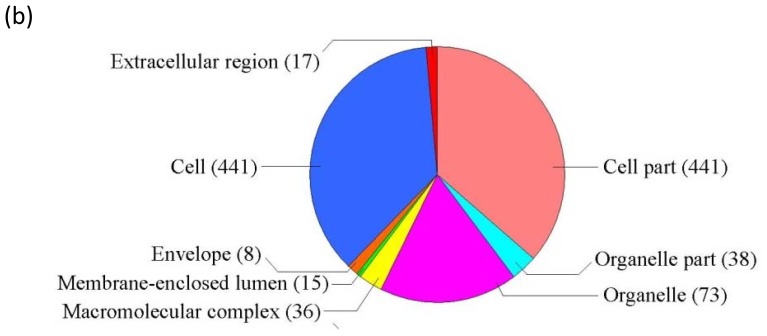
GO term pie chart of “Cellular component” for NaCl- and ABA-stressed genes. (**a**) Indicates GO term pie picture of “Cellular component” under NaCl-stressed genes; (**b**) Indicates GO term pie picture of “Cellular component” under ABA-stressed genes.

**Figure 3:**
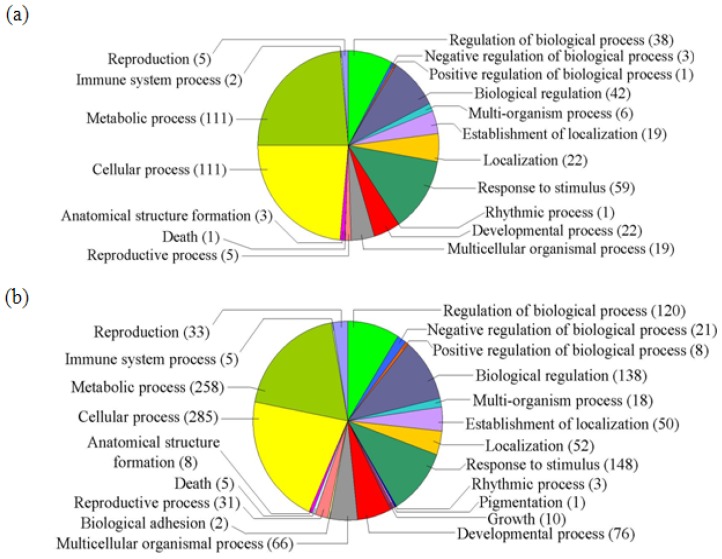
GO term pie chart of “Biological process” for NaCl- and ABA-stressed gene samples. (**a**) Indicates GO term pie picture of “Biological process” under NaCl-stressed genes; (**b**) Indicates GO term pie picture of “Biological process” under ABA-stressed genes.

GO classification was conducted for the pathways significantly (*p* < 0.05) enriched in the differentially regulated genes under salt stress. Among the “Molecular function” group, 108 genes were in the “Catalytic activity” category, while 92 genes were “Binding function” genes, 33 genes were “Transcription regulator activity factor” genes, and 16 genes were in the “Transporter activity” category (Figure 1a). These results suggest that these pathways are highly activated under salt stress. For the GO term “Cellular component”, 165 genes were categorized as “Cell” and “Cell part” component genes, while 73 and 14 genes belong to “Organelle” and “Organelle part”, respectively, and eight genes belong to “Macromolecular complex” (Figure 2a). Numerous genes were classified into the “Biological process” category, including 111, 111, 59, 42, 38, 22 and 19 genes in the categories “Cellular process”, “Metabolic process”, “Response to stimulus”, “Biological regulation”, “Regulation of biological process”, “Developmental process” and “Multicellular organismal process”, respectively (Figure 3a). The genes involved in these pathways were highly accumulated, indicating that these pathways are important for the salt stress response.

### 2.2. Genes Expressed in Response to ABA Treatment

To identify genes expressed in *Arabidopsis* in response to ABA stress, gene expression in plants treated with 10 μM ABA for 6 h before harvest was compared with that of control plants using Agilent GeneChip microarray analysis. With *P*-value > 0.05 and a threshold of twofold change, we determined that 408 genes were significantly upregulated, and 381 genes were significantly downregulated under ABA stress conditions (Supporting Information Table S2). In our study, however, we were not able to obtain RD29A and COR15A, the marker genes response to ABA signal in the study of Goda *et al.* (2008) [45]. Nevertheless, some other marker genes which play an important role in responding to ABA were monitored in the profile, such as ABIs and AREB/ABFs [46,47], the governor of ABA signal-regulated genes, where bZIP transcription factors were observed in our study [48,49]. In addition, there are many genes related to stress tolerance that were upregulated by ABA treatment, including genes encoding LEA family proteins, WRKY and MYB transcription factors, leucine zipper proteins, glutathione S-transferases (GST), nc RNA and ERF transcription factors, all suggesting that these genes are involved in the ABA signaling stress response.

To study whether these differential expression genes are regulated by bZIP transcription factors via binding to the ABA-responsive-element (ABRE) in their promoter, we randomly selected the genes which upregulated more than fivefold under ABA stress treatment, and screened their promoter region by searching ABRE (5′-CACGTG-3′) motifs. The results showed that almost all of these genes have at least one motif of ABRE in their promoter region and most of them have two or more ABRE motifs (Supporting Information Table S3). This result confirmed that these genes are involved in the ABA signaling stress response, regulated by bZIP transcription factors via binding to ABRE in their promoter.

GO analysis was conducted for the pathways significantly *(P* < 0.05) enriched in the differentially expressed genes under ABA treatment. In the “Molecular function” category, there were 242 genes belonging to “Catalytic activity”, 285 genes belonging to “Binding function”, 88 genes involved in “Transcription regulator activity factor”, and 37 genes involved in “Transporter activity.” These functional pathways are highly induced by ABA and play important roles in ABA-dependent signaling pathways. In addition, the subgroup “Molecular transducer activity”, which contains 11 genes, was differentially regulated by ABA but not salt stress (Figure 1b), suggesting that this pathway is important for the ABA-dependent signaling pathway. In the term “Cellular component”, 441 genes were grouped into the “Cell” and “Cell part” components, while 213 and 38 genes were categorized as “Organelle” and “Organelle part” component genes, respectively, and 36 genes were categorized as “Macromolecular complex” genes (Figure 2b).

In the “Biological process” category, there were 285, 258, 148, 138, 120, 76 and 66 genes grouped into “Cellular process”, “Metabolic process”, “Response to stimulus”, “Biological regulation”, “Regulation of biological process”, “Developmental process” and “Multicellular organismal process”, respectively (Figure 3b). These results indicate that these processes play important roles in ABA-dependent signaling pathway. Moreover, 10 genes in the “Growth” category were differentially regulated by ABA but not NaCl stress, suggesting that this process is involved in the ABA-dependent signaling pathway but is not involved in the salt stress response.

### 2.3. Confirmation of Microarray Results by Quantitative Real-Time PCR

**Figure 4:**
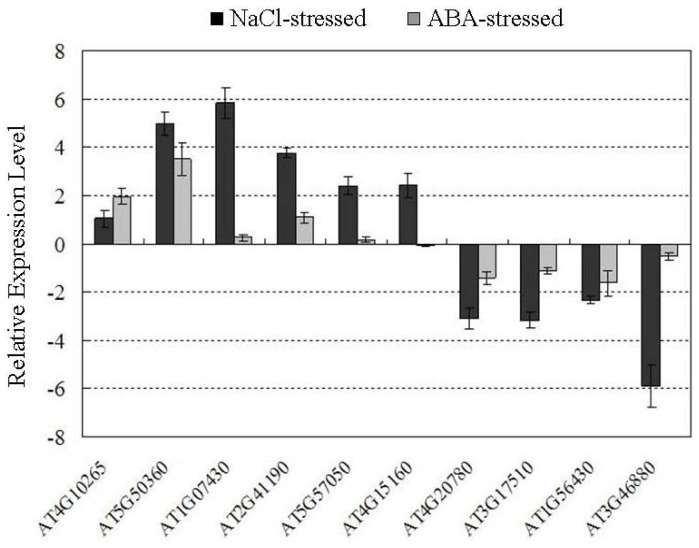
Expression analysis of genes under NaCl and ABA stresses.

In the microarray analysis, saturation of fluorescent signals in the microarray or to cross-hybridization among genes is possible to occur, which leads to false results; therefore, we performed quantitative real-time PCR analysis on specific transcripts to provide further validation of our microarray experiment data. We selected 10 genes representing different functional categories in response to NaCl and ABA treatment according to our microarray analysis, including: five genes that have more expression than others and were upregulated by both stressors; four genes which have less expression than others and were downregulated by both stressors; and one gene that was upregulated by NaCl stress, but downregulated by ABA stress (Figure 4). All 10 of the genes showed the same expression patterns with quantitative real-time PCR as they did with microarray analysis, demonstrating the reliability of the microarray results. However, the fold-changes determined by microarray analysis and real-time RT-PCR were slightly different (Table 1). This may have been due to technical differences in the analysis and normalization methods.

### 2.4. Comparison of Gene Expression Profiles between Salt and ABA Treatments

**Figure 5:**
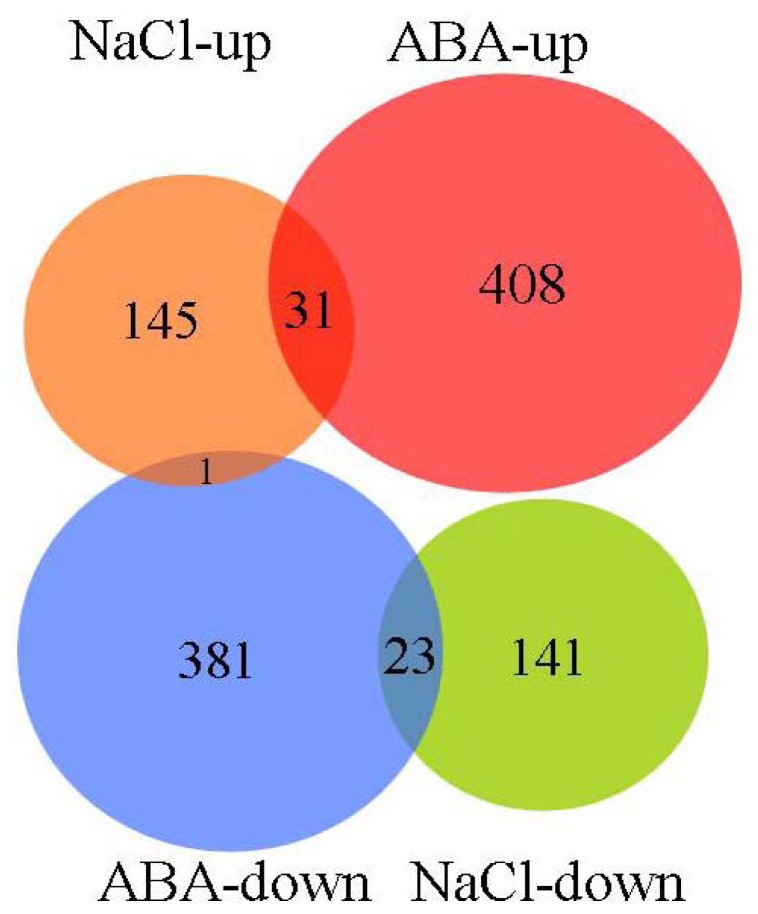
Venn diagram for the proportions of genes affected by NaCl stress and ABA stress. “NaCl-up” and “NaCl-down” indicate genes up- and downregulated, respectively, under NaCl treatment; “ABA-up” and “ABA-down” indicate genes up- and downregulated, respectively, under ABA treatment.

We further compared the levels of gene expression in response to salt and ABA treatment. The results show that ABA regulates the expression of more genes than salt stress. Among the differentially expressed genes, 31 and 23 genes were upregulated and downregulated, respectively, in both NaCl- and ABA-stressed plants. Only one gene showed opposite patterns of regulation in response to the two treatments (Figure 5).

**Figure 6:**
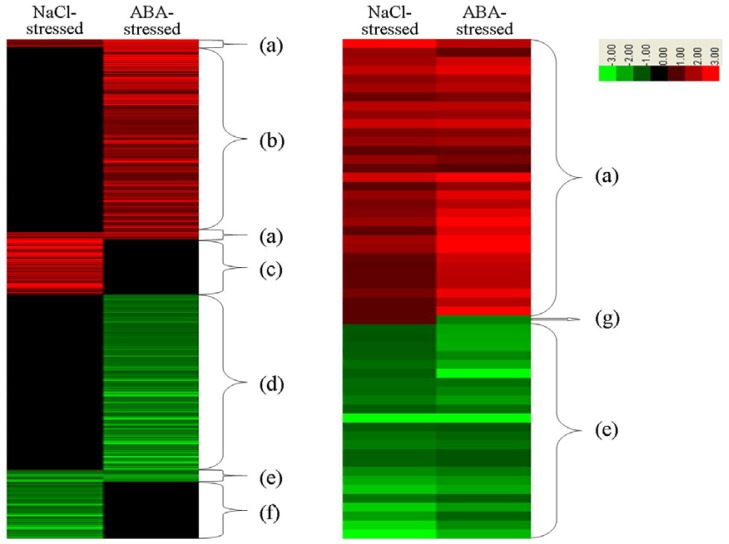
Microarray analysis of NaCl- and ABA stresses. Part (**a**) indicates both upregulated genes by both NaCl and ABA stresses; part (**b**) indicates genes are upregulated by ABA only; part (**c**) indicates genes are upregulated by NaCl stress only; part (**d**) indicates genes are downregulated by ABA stress only; part (**e**) indicates both downregulated patterns by NaCl and ABA stresses; part (**f**) indicates genes are downregulated by NaCl stress only.

Genes that were differentially regulated by salt or ABA treatment were classified into categories according to their expression patterns (Figure 6). A total of 33 genes belong to group (a) (upregulated by both NaCl and ABA stress), which only accounted for a small proportion of the differentially expressed genes (Table 2), including genes encoding proteins such as transcription factor NAC19, NAC72, bHLH122, ATHB21 and KNAT3 and protein phosphatase 2C-3. Prominently, there were eight genes containing one or more ABRE element in their promoter able to regulate by bZIP transcription factors. The results indicated that those genes indeed can be directly involved in ABA dependent regulation. In addition, 23 genes comprise group (e), *i.e*., genes that were downregulated by both NaCl and ABA stresses. This group also accounted for only a small proportion of differentially expressed genes (Table 3), including genes encoding transcription factor BZIP3 and WOX2, ubiquitin–protein ligase UBC17, heat shock protein class V15.4, sulfate adenylyltransferase APS3, calcium-binding protein CML42, IDA-like 5 protein, and others. Many genes belonging to group (a) and group (e) have been shown to function in multiple stress and ABA responses. Therefore, genes that are up- or downregulated by both ABA and salt may play common roles in the salt and ABA stress responses. However, there is only one differentially expressed gene in group (g) (upregulated by NaCl stress but downregulated by ABA stress), which encodes an LTP family protein, which showed opposite expression patterns in response to NaCl and ABA stress (Table 4). LTPs, which are pathogenesis-related (PR) proteins, are probably involved in the pathogen defense response in plants [35]. Obviously, this LTP gene is a stress-responsive gene that is regulated by ABA and other stress signals, resulting in the differentially expression pattern of this gene in plants exposed to salt or ABA stress. Unfortunately, there is no differential gene that was downregulated under NaCl stress while upregulated under ABA stress.

**Figure 7:**
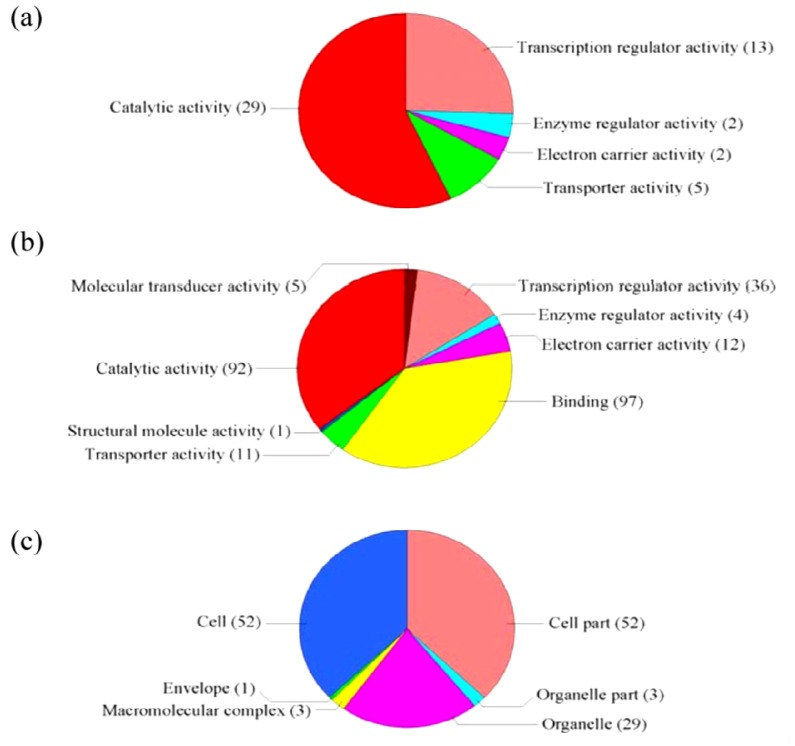

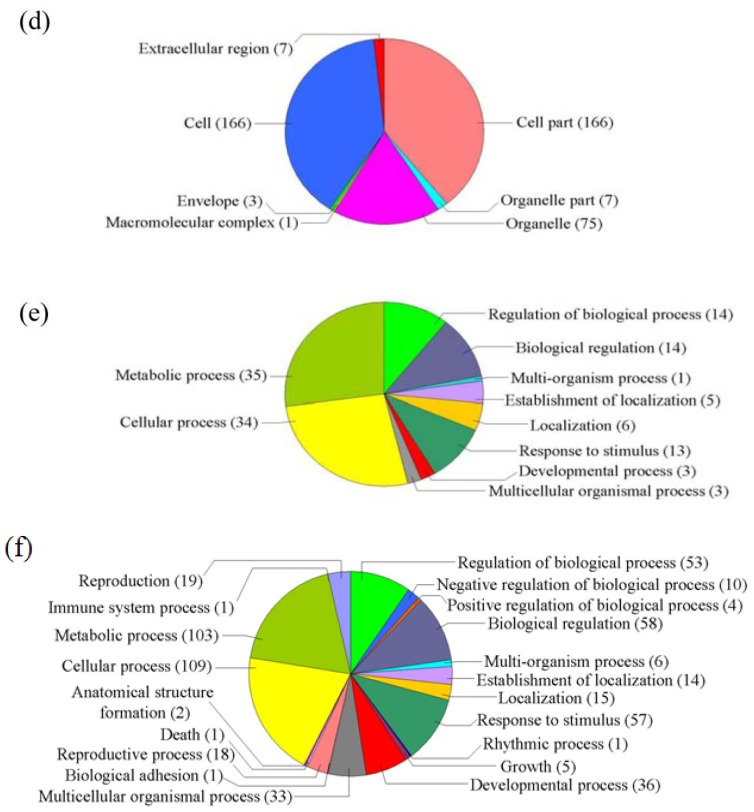
GO term pie chart of the only differently upregulated genes from NaCl and ABA stresses. (**a**) and (**b**) indicates GO term pie picture of “Molecular function” for the only upregulated genes under NaCl and ABA stresses, respectively; (**c**) and (**d**) indicates GO term pie picture of “Cellular component” for the only upregulated genes under NaCl and ABA stresses, respectively; (**e**) and (**f**) indicates GO term pie picture of “Biological process” for the only upregulated genes for the NaCl- and ABA-stressed samples, respectively.

**Figure 8:**
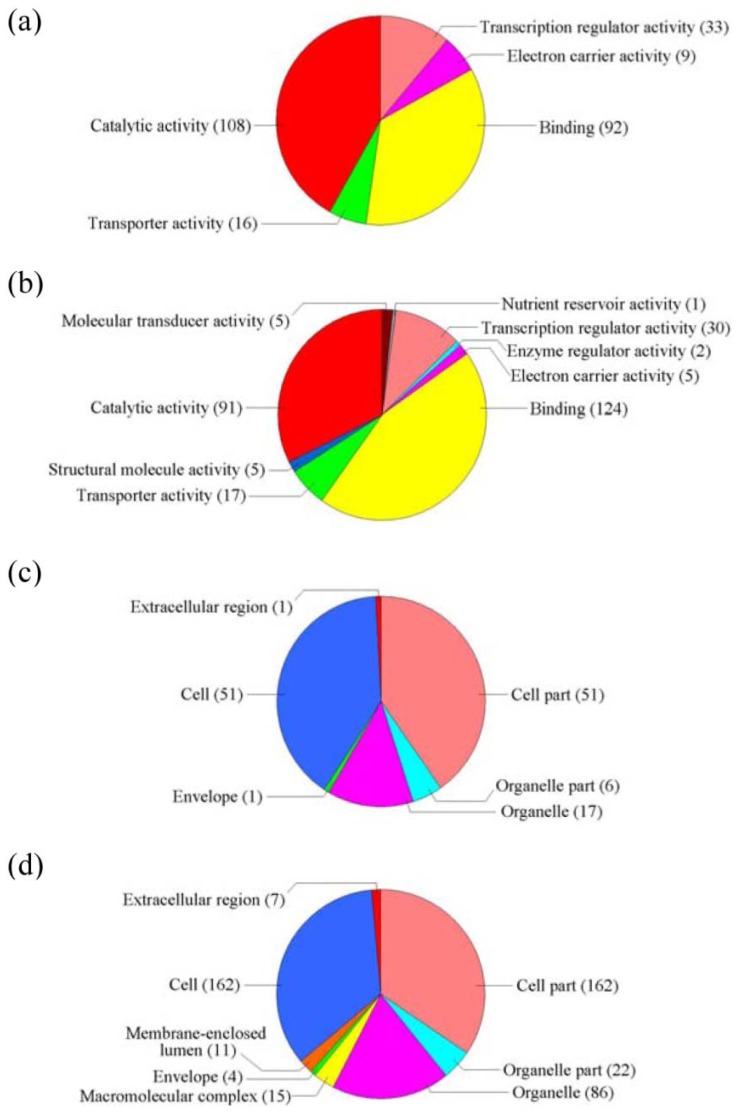

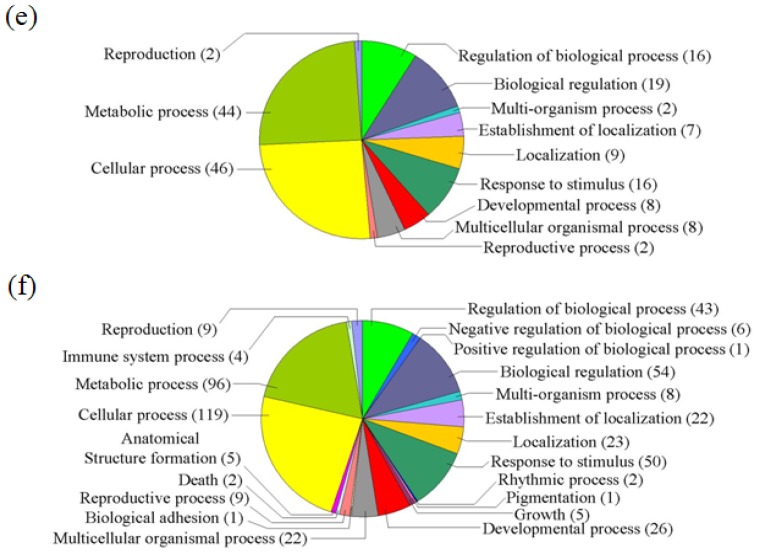
GO term pie chart of the only differently downregulated genes under NaCl and ABA stresses. (**a**) and (**b**) indicates GO term pie picture of “Molecular function” for the only downregulated genes in the NaCl- and ABA-stressed samples, respectively; (**c**) and (**d**) indicates GO term pie picture of “Cellular component” for the only downregulated genes from NaCl and ABA stresses, respectively; (**e**) and (**f**) indicates GO term pie picture of “Biological process” for the only downregulated genes under NaCl and ABA stresses, respectively.

There were many more genes in group (b) (upregulated by ABA but not altered by NaCl) and group (d) (downregulated by ABA but not altered by NaCl) than in group (c) (upregulated by NaCl but not altered by ABA) and group (f) (downregulated by NaCl stress but not altered by ABA). These results suggest that ABA regulates the expression of more genes than salt stress. GO analysis of the differentially regulated genes in group (b) and group (c) revealed that many functional pathways are involved in ABA stress but not salt stress (Figure 7). For instance, genes in the GO categories “Molecular function” and “Cellular component”, subgroups “Molecular transducer”, “Binding”, “Structural molecule activity”, “Molecular transducer”, “Nutrient reservoir activity”, “Enzyme regulator activity”, “Structural molecule activity”, “Macromolecular complex” and “Extracellular region” were differentially regulated by ABA stress but not NaCl stress. In addition, genes in the GO categories “Biological processes”, “Negative regulation of biological process”, “Negative regulation of biological process”, “Rhythmic process”, “Growth”, “Reproductive process”, “Death”, “Anatomical structure formation”, “Biological adhesion”, “Immune system process”, “Reproduction” and “Biological adhesion” were induced by ABA but not NaCl (Figure 8). These results suggest that ABA treatment regulates the expression of more genes than salt stress. This is an important phenomenon that deserves further investigation.

## 3. Experimental Section

### 3.1. Plant Material and Growth Conditions

*Arabidopsis thaliana* ecotype Columbia (Col-0) was used for this experiment. Four-week-old seedlings were grown under a natural photoperiod in freshwater-irrigated pots and were treated with either 150 mM NaCl or 10 μM ABA for 6 hours. Unstressed seedlings (control sample) were collected in parallel to avoid the possible effects of circadian rhythms. The whole seedlings including root and shoot were harvested and pooled for each treatment before freezing in liquid nitrogen and storage at −80 °C for RNA isolation. The harvested seedlings in each treatment were divided into three portions as independent biological replicates.

### 3.2. RNA Extraction and Reverse Transcription

Total RNA was extracted from each sample using Trizol reagent. After processing the RNA with DNase (Promega, Madison, WI, USA) digestion and cleanup procedures, the total RNA concentration was examined using a NanoVue Plus Spectrophotometer, followed by storage at −80 °C until further analysis.

A total of 500 ng of DNase-treated RNA was reverse transcribed in a 10 μL volume of reaction mixture using a PrimeScript^TM^ RT Reagent Kit following the manufacturer’s protocol (TaKaRa, Kyoto, Japan). The procedure was performed using reverse transcriptase with oligodeoxythymidine primer and six random primers at 37 °C for 45 min, followed by deactivation of the reverse transcriptase at 80 °C for 5 s. PCR was performed with a 10-fold dilution of cDNA reaction mixture in a final volume of 20 μL using rTaq DNA polymerase (TaKaRa, Kyoto, Japan).

### 3.3. Microarray Analysis

To identify differences in gene expression between *Arabidopsis* treated with NaCl and ABA stress, Agilent *Arabidopsis* oligo microarray analysis was employed. Two-μg aliquots of total RNA from NaCl-treated and ABA-treated plants were prepared and microarray hybridized using the Gene-Chip^®^ 3′IVT Express Kit (Agilent) and Gene-Chip^®^ hybridization. After hybridization, the microarray slides were washed and stained according to the manufacturer’s standard protocol (Agilent). Normalization of all arrays was performed by quantify normalization using MAS 5.0 to standardize the distribution of probe intensities for each array in a set of arrays [50,51]. The differentially expressed genes (up- or downregulated) between the NaCl-stressed and ABA-stressed plants were selected with a significance *P*-value of <0.05 and analyzed using Welch’s *t*-test [52]. Gene ontology (GO) terms for genes that were differentially expressed under NaCl and ABA treatment were divided into three levels: molecular function, cellular component and biological process.

### 3.4. Verification of Microarray Data

Real-time quantitative PCR was used to validate selected data from the microarray experiments and to examine the expression of a subset of genes over time. The genes and primers used for the RT-PCR assays are listed in Table 5. The cDNA products were diluted and used for quantitative real-time PCR analysis, which was performed using a DNA Engine OPTICON 2 Continuous Fluorescence Detector with SYBR-Green Real-time PCR Master Mix (Toyobo, Co., Ltd., Tokyo, Japan) according to the manufacturer’s instructions. Amplification conditions were as follows: hot start at 94 °C for 30 s followed by 45 cycles of 12 s at 94 °C, 30 s at 60 °C, 40 s at 70 °C and 1 s at 80 °C for plate reading. The *Arabidopsis* ubiquitin gene and á-tublin gene were used as the endogenous control, and each sample was run side by side in triplicate. After completion of PCR amplification, all of the data were analyzed using Opticon Monitor 2 software (ABI) based on the 2^−ΔΔCt^ method [53].

## 4. Conclusions

In summary, we identified genes that were differentially regulated in response to salt and ABA stress. Some of these genes were up- or downregulated by ABA and salt stress, but ABA triggered or inhibited the expression of more genes than salt stress. Furthermore, GO analysis also revealed that many processes are involved in the ABA-dependent signaling pathway but are not involved in the salt stress response. The results indicate that these processes play important roles in the ABA-dependent signaling pathway. Our results may help elucidate differences in the response of plants to salt and ABA stress.

## Figures and Tables

**Table 1:** Real-time quantitative RT-PCR.

seq	Locustag	Annotation	Microarray NaCl-logFC	RT-PCR NaCl-logFC	Microarray ABA-logFC	RT-PCR ABA-logFC
1	AT4G10265	putative wound-responsive protein	4.8003	1.0353	2.1751	1.959
2	AT5G50360	hypothetical protein	2.5511	4.973	4.0022	3.5212
3	AT1G07430	protein phosphatase 2C 3	1.8292	5.835	4.2210	0.2495
4	AT2G41190	transmembrane amino acid transporter-like protein	1.7845	3.7497	3.6432	1.0875
5	AT5G57050	ABI2	1.0575	2.4057	3.4208	0.1722
6	AT4G15160	LTP family protein	1.0653	2.421	−1.5312	−0.0905
7	AT4G20780	calcium-binding protein CML42	−1.0333	−3.0587	−1.9896	−1.4238
8	AT3G17510	CBL-interacting serine	−1.1578	−3.153	−1.0133	−1.1058
9	AT1G56430	nicotianamine synthase	−1.8847	−2.319	−1.1996	−1.6192
10	AT3G46880	protein coding	−3.6012	−5.8873	−4.0783	−0.5008

**Table 2:** Common upregulated genes under NaCl and ABA stresses.

Locustag	NaCl-stressed fold change	ABA-stressed fold change	ABRE element	Annotation
AT4G10265	27.8632	4.5162	–	wound-responsive protein
AT5G50360	5.8608	16.0246	−413 (CACGTG) −78 (CACGTG)	hypothetical protein
AT5G49120	5.3675	5.7876	−177 (CACGTG)	senescence-associated protein-related
AT5G40790	4.8776	6.6758	−158 (CACGTG)	hypothetical protein
AT5G53710	4.6116	4.6627	–	hypothetical protein
AT1G52890	3.8061	4.5065	−164 (CACGTG) −794 (CACGTG) −993 (CACGTG)	transcription factor NAC19
AT5G63350	3.7261	16.2454	–	hypothetical protein
AT2G18550	3.7087	5.5151	–	leucine zipper protein ATHB-21, transcription factor
AT1G07430	3.5534	18.6473	–	protein phosphatase 2C-3
AT1G47960	3.4988	2.2437	–	vacuolar inhibitor of fructosidase 1
AT4G02280	3.4619	3.5309	–	UDP-glycosyltransferase
AT2G41190	3.4442	12.4944	–	transmembrane amino acid transporter-like protein
AT4G14819	3.4372	3.7987	–	hypothetical protein
AT5G53120	3.4023	2.7388	–	SPDS3 (spermine synthase 3)
AT3G22560	3.3933	6.3729	–	GNAT family protein
AT3G48020	3.1747	4.0543	–	protein coding
AT1G80160	2.9702	6.5128	−369 (CACGTG) −153 (CACGTG)	Lactoylglutathione lyase
AT5G40690	2.8515	3.0216	–	hypothetical protein
AT2G25625	2.6942	4.4558	–	hypothetical protein
AT4G18980	2.6594	7.0188	–	protein AtS40-3
AT4G27410	2.4024	2.7636	–	transcription factor NAC72
AT3G11410	2.2645	5.3254	−397 (CACGTG)	protein phosphatase 2C-37
AT3G14360	2.2635	2.0886	–	lipase class 3 family protein
AT1G79520	2.2204	4.6101	–	Cation efflux family protein
AT5G17460	2.2132	4.5499	–	hypothetical protein
AT2G39800	2.1813	6.0887	−209 (CACGTG)	Gamma-glutamyl phosphate reductase
AT5G25220	2.1642	2.2841	–	transcription factor KNAT3
AT1G17940	2.1421	4.8303	–	BRO1-like domain-containing protein
AT1G51140	2.1383	3.3635	−284 (CACGTG)	transcription factor bHLH122
AT5G66400	2.1236	4.5373	–	dehydrin RAB18
AT5G57050	2.0813	10.7091	–	ABI2

**Table 3:** Common downregulated genes under NaCl and ABA stresses.

Locustag	NaCl-stressed fold change	ABA-stressed fold change	Annotation
AT4G20780	0.4886	0.2518	calcium-binding protein CML42
AT3G03780	0.4726	0.2624	threonine-protein kinase WNK-related protein
AT4G37670	0.4649	0.2412	*N*-acetyl-l-glutamate synthase 2
AT2G23600	0.4647	0.4048	acetone-cyanohydrin lyase
AT5G59080	0.4621	0.497	hypothetical protein
AT4G14680	0.4594	0.3298	sulfate adenylyltransferase APS3
AT4G21870	0.458	0.1104	heat shock protein class V 15.4
AT3G17510	0.4482	0.4954	CBL-interacting serine
AT5G19230	0.4458	0.4943	GPI-anchored glycoprotein membrane precursor
AT1G76952	0.4207	0.3389	IDA-like 5 protein
AT4G36410	0.4118	0.3982	ubiquitin-protein ligase UBC17
AT1G33340	0.4064	0.2444	clathrin assembly protein
AT1G55380	0.3985	0.4387	histidine-rich C1 domain-containing protein
AT5G15830	0.3832	0.464	transcription factor BZIP3
AT5G59340	0.3664	0.2786	transcription factor WOX2
AT1G58370	0.3592	0.3879	glycosyl hydrolase-like prottein 10
AT1G76990	0.3184	0.3688	ACT domain-containing protein 3
AT1G56430	0.2708	0.4354	nicotianamine synthase
AT5G04310	0.2456	0.291	pectate lyase family protein
AT5G57220	0.1931	0.2487	CYP81F2
AT3G15356	0.1843	0.2811	lectin-like protein
AT3G16530	0.1668	0.3124	legume lectin-like protein
AT3G46880	0.0824	0.0592	protein coding

**Table 4:** Upregulated gene under NaCl stress, while downregulated under ABA stress.

Locustag	NaCl-stressed fold change	ABA-stressed fold change	Annotation
AT4G15160	2.0926	0.346	LTP family protein

**Table 5:** Primer used for real-time quantitative RT-PCR.

seq	Locus tag	Forward Primers (5′-3′)	Reverse Primers (5′-3′)
1	AT4G10265	ATGAGCTCTGCAAGCAAAACGT	TTAACTAGGACCCCAACAGCTC
2	AT5G50360	ATCAGACAGAGTTAATGACGG	CATCACAGAACGAAGTAATCG
3	AT1G07430	ATGTCACGAGCCATAGGAGAC	AGCTTCGTCAGCAATACTGACG
4	AT2G41190	ATTACACTAAACATGCCACAGG	CAATGATCGAACTCAGAATCAT
5	AT5G57050	ATGAAGCGGCGAGGATAGAAGCTG	CTGCCGCGGACATTGCTGCAGGAT
6	AT4G15160	ACTGTGAAGCCACCACCACCTC	CGTCAAGACCCACTAAGTCGTC
7	AT4G20780	AACGCTGATCTCTCCGATCTC	GATTCCGGTCAACGGAAACGAT
8	AT3G17510	AGTACATTCCTTCAATTCCCGATG	CAGCTGTTACTGATAAACCAACTT
9	AT1G56430	ATCGACCCATCAGCGAATATGGT	CGAAGCTTGATCTATATGATCAT
10	AT3G46880	ATGGAGGGTAAAGGAAGAGTTG	CATCGTTGTTATCTCTGTTCTGAT

## References

[1] DeRisi J., Penland L., Brown P.O., Bittner M.L., Meltzer P.S., Ray M., Chen Y., Su Y.A., Trent J.M. (1996). Use of a cDNA microarray to analyse gene expression patterns in human cancer. Nat. Genet.

[2] Kilian J., Peschke F., Berendzen K.W., Harter K., Wanke D. (2012). Prerequisites, performance and profits of transcriptional profiling the abiotic stress response. Biochim. Biophys. Acta.

[3] Redman J.C., Haas B.J., Tanimoto G., Town C.D. (2004). Development and evaluation of an Arabidopsis whole genome Affymetrix probe array. Plant J.

[4] Zeller G., Henz S.R., Widmer C.K., Sachsenberg T., Ratsch G., Weigel D., Laubinger S. (2009). Stress-induced changes in the Arabidopsis thaliana transcriptome analyzed using whole-genome tiling arrays. Plant J.

[5] Kaya C., Ilktac A., Gokmen E., Ozturk M., Karaman I.M. (2007). The long-term results of transurethral vaporization of the prostate using plasmakinetic energy. BJU Int.

[6] Allakhverdiev S.I., Sakamoto A., Nishiyama Y., Inaba M., Murata N. (2000). Ionic and osmotic effects of NaCl-induced inactivation of photosystems I and II in Synechococcussp. Plant Physiol.

[7] Chinnusamy V., Jagendorf A., Zhu J.K. (2005). Understanding and improving salt tolerance in plants. Crop Sci.

[8] Maricle B.R., Cobos D.R., Campbell C.S. (2007). Biophysical and morphological leaf adaptations to drought and salinity in salt marsh grasses. Environ. Exp. Bot.

[9] Sekmen A.H., Turkan I., Takio S. (2007). Differential responses of antioxidative enzymes and lipid peroxidation to salt stress in salt-tolerant Plantago maritima and salt-sensitive Plantago media. Physiol. Plant.

[10] Kreps J.A., Wu Y., Chang H.-S., Zhu T., Wang X., Harper J.F. (2002). Transcriptome changes for Arabidopsis in response to salt, osmotic, and cold stress. Plant Physiol.

[11] Kilian J., Whitehead D., Horak J., Wanke D., Weinl S., Batistic O., D’Angelo C., Bornberg-Bauer E., Kudla J., Harter K. (2007). The AtGenExpress global stress expression data set: Protocols, evaluation and model data analysis of UV-B light, drought and cold stress responses. Plant J.

[12] Watanabe S., Kojima K., Ide Y., Sasaki S. (2000). Effects of saline and osmotic stress on proline and sugar accumulation in Populus euphratica *in vitro*. Plant Cell Tissue Organ Cult.

[13] Ranf S., Wünnenberg P., Lee J., Becker D., Dunkel M., Hedrich R., Scheel D., Dietrich P. (2008). Loss of the vacuolar cation channel, AtTPC1, does not impair Ca^2+^ signals induced by abiotic and biotic stresses. Plant J.

[14] Jiang Y., Deyholos M.K. (2006). Comprehensive transcriptional profiling of NaCl-stressed Arabidopsis roots reveals novel classes of responsive genes. Plant Biol.

[15] Seki M., Narusaka M., Ishida J., Nanjo T., Fujita M., Oono Y., Kamiya A., Nakajima M., Enju A., Sakurai T. (2002). Monitoring the expression profiles of 7000 Arabidopsis genes under drought, cold and high-salinity stresses using a full-length cDNA microarray. Plant J.

[16] Takahashi S., Seki M., Ishida J., Satou M., Sakurai T., Narusaka M., Kamiya A., Nakajima M., Enju A., Akiyama K. (2004). Monitoring the expression profiles of genes induced by hyperosmotic, high salinity, and oxidative stress and abscisic acid treatment in Arabidopsis cell culture using a full-length cDNA microarray. Plant Mol. Biol.

[17] Kawasaki S., Borchert C., Deyholos M., Wang H., Brazille S., Kawai K., Galbraith D., Bohnert H.J. (2001). Gene expression profiles during the initial phase of salt stress in rice. Plant Cell.

[18] Rabbani M.A., Maruyama K., Abe H., Khan M.A., Katsura K., Ito Y., Yoshiwara K., Seki M., Shinozaki K., Yamaguchi-Shinozaki K. (2003). Monitoring expression profiles of rice genes under cold, drought, and high-salinity stresses and abscisic acid application using cDNA microarray and RNA get-blot analyses. Plant Physiol.

[19] Adie B.A., Perez-Perez J., Perez-Perez M.M., Godoy M., Sanchez-Serrano J.J., Schmelz E.A., Solano R. (2007). ABA is an essential signal for plant resistance to pathogens affecting JA biosynthesis and the activation of defenses in Arabidopsis. Plant Cell.

[20] Finkelstein R.R., Gampala S.S., Rock C.D. (2002). Abscisic acid signaling in seeds and seedlings. Plant Cell.

[21] Cheong Y.H., Moon B.C., Kim J.K., Kim C.Y., Kim M.C., Kim I.H., Park C.Y., Kim J.C., Park B.O., Koo S.C. (2003). BWMK1, a rice mitogen-activated protein kinase, locates in the nucleus and mediates pathogenesis-related gene expression by activation of a transcription factor. Plant Physiol.

[22] Kang J.Y., Choi H.I., Im M.Y., Kim S.Y. (2002). Arabidopsis basic leucine zipper proteins that mediate stress-responsive abscisic acid signaling. Plant Cell.

[23] Fujita Y., Fujita M., Shinozaki K., Yamaguchi-Shinozaki K. (2011). ABA-mediated transcriptional regulation in response to osmotic stress in plants. J. Plant Res.

[24] Cutler S.R., Rodriguez P.L., Finkelstein R.R., Abrams S.R. (2010). Abscisic acid: Emergence of a core signaling network. Annu. Rev. Plant Biol.

[25] Xiong L., Schumaker K.S., Zhu J.K. (2002). Cell signaling during cold, drought, and salt stress. Plant Cell.

[26] Zhang J., Jia W., Yang J., Ismail A.M. (2006). Role of ABA in integrating plant responses to drought and salt stresses. Field Crops Res.

[27] Yamaguchi-Shinozaki K., Shinozaki K. (2006). Transcriptional regulatory networks in cellular responses and tolerance to dehydration and cold stresses. Annu. Rev. Plant Biol.

[28] Chinnusamy V., Schumaker K., Zhu J.K. (2004). Molecular genetic perspectives on cross-talk and specificity in abiotic stress signalling in plants. J. Exp. Bot.

[29] Thomashow M.F. (1999). Plant cold acclimation: Freezing Tolerance Genes and Regulatory Mechanisms. Annu. Rev. Plant Physiol. Plant Mol. Biol.

[30] Shinozaki K., Yamaguchi-Shinozaki K. (1997). Gene expression and signal transduction in water-stress response. Plant Physiol.

[31] Mahajan S., Tuteja N. (2005). Cold, salinity and drought stresses: An overview. Arch. Biochem. Biophys.

[32] Uno Y., Furihata T., Abe H., Yoshida R., Shinozaki K., Yamaguchi-Shinozaki K. (2000). Arabidopsis basic leucine zipper transcription factors involved in an abscisic acid-dependent signal transduction pathway under drought and high-salinity conditions. Proc. Natl. Acad. Sci. USA.

[33] Nakashima K., Shinwari Z.K., Sakuma Y., Seki M., Miura S., Shinozaki K., Yamaguchi-Shinozaki K. (2000). Organization and expression of two Arabidopsis DREB2 genes encoding DRE-binding proteins involved in dehydration- and high-salinity-responsive gene expression. Plant Mol. Biol.

[34] Sharma N., Abrams S.R., Waterer D.R. (2005). Uptake, movement, activity, and persistence of an abscisic acid analog (8′Acetylene ABA methyl ester) in marigold and tomato. J. Plant Growth Regul..

[35] Bohra J.S., Dörffling H., Dörffling K. (1995). Salinity tolerance of rice (Oryza sativa L.) with reference to endogenous and exogenous abscisic acid. J. Agron. Crop Sci.

[36] Wilkinson S., Davies W.J. (2002). ABA-based chemical signal-ling: The co-ordination of responses to stress in plants. Plant Cell Environ.

[37] Christmann A., Weiler E.W., Steudle E., Grill E. (2007). A hydraulic signal in root-to-shoot signalling of water shortage. Plant J.

[38] Yokoi S., Quintero F.J., Cubero B., Ruiz M.T., Bressan R.A., Hasegawa P.M., Pardo J.M. (2002). Differential expression and function of Arabidopsis thaliana NHX Na+/H+ antiporters in the salt stress response. Plant J.

[39] Saeedipour S. (2011). Salinity tolerance of rice lines related to endogenous abscisic acid (ABA) level synthesis under stress. Afr. J. Plant Sci.

[40] Xiong L., Zhu J.K. (2003). Regulation of abscisic acid biosynthesis. Plant Physiol.

[41] Zhu J.K. (2002). Salt and drought stress signal transduction in plants. Annu. Rev. Plant Biol.

[42] Yamaguchi-Shinozaki K., Shinozaki K. (1993). Characterization of the expression of a desiccation-responsive rd29 gene of Arabidopsis thaliana and analysis of its promoter in transgenic plants. Mol. Gen. Genet.

[43] Yamaguchi-Shinozaki K., Shinozaki K. (1993). The plant hormone abscisic acid mediates the drought-induced expression but not the seed-specific expression of rd22, a gene responsive to dehydration stress in Arabidopsis thaliana. Mol. Gen. Genet.

[44] Fujita M., Fujita Y., Maruyama K., Seki M., Hiratsu K., Ohme-Takagi M., Tran L.S., Yamaguchi-Shinozaki K., Shinozaki K. (2004). A dehydration-induced NAC protein, RD26, is involved in a novel ABA-dependent stress-signaling pathway. Plant J.

[45] Goda H., Sasaki E., Akiyama K., Maruyama-Nakashita A., Nakabayashi K., Li W., Ogawa M., Yamauchi Y., Preston J., Aoki K. (2008). The AtGenExpress hormone and chemical treatment data set: Experimental design, data evaluation, model data analysis and data access. Plant J.

[46] Bossi F., Cordoba E., Dupré P., Mendoza M.S., Román C.S., León P. (2009). The Arabidopsis ABA-INSENSITIVE (ABI) 4 factor acts as a central transcription activator of the expression of its own gene, and for the induction of ABI5 and SBE2.2 genes during sugar signaling. Plant J.

[47] Lee S.J., Kang J.Y., Park H.J., Kim M.D., Bae M.S., Choi H.I., Kim S.Y. (2010). DREB2C interacts with ABF2, a bZIP protein regulating abscisic acid-responsive gene expression, and its overexpression affects abscisic acid sensitivity. Plant Physiol.

[48] Lu G., Paul A.L., McCarty D.R., Ferl R.J. (1996). Transcription factor veracity: Is GBF3 responsible for ABA-regulated expression of Arabidopsis Adh?. Plant Cell.

[49] Chen W.Q., Provart N.J., Glazebrook J., Katagiri F., Chang H.S., Eulgem T., Mauch F., Luan S., Zou G.Z., Whitham S.A. (2002). Expression profile matrix of Arabidopsis transcription factor genes suggests their putative functions in response to environmental stresses. Plant Cell.

[50] Wang Y., Li H., Si Y., Zhang H., Guo H., Miao X. (2012). Microarray analysis of broad-spectrum resistance derived from an indica cultivar Rathu Heenati. Planta.

[51] Jia M.A., Li Y., Lei L., Di D., Miao H., Fan Z. (2012). Alteration of gene expression profile in maize infected with a double-stranded RNA fijivirus associated with symptom development. Mol. Plant Pathol.

[52] Wolfinger R.D., Gibson G., Wolfinger E.D., Bennett L., Hamadeh H., Bushel P., Afshari C., Paules R.S. (2001). Assessing gene significance from cDNA microarray expression data via mixed models. J. Comput. Biol.

[53] Livak K.J., Schmittgen T.D. (2001). Analysis of relative gene expression data using real-time quantitative PCR and the 2(-Delta Delta C(T)) Method. Methods.

